# *Gryphopsylla segregata* Beaucournu & Sountsov, 1999 : Nouveau statut pour *G. jacobsoni segregata*, description de la femelle et proposition d’une nouvelle clé pour le genre *Gryphopsylla* traub, 1957 (Siphonaptera – Pygiopsyllidae – Stivaliinae)

**DOI:** 10.1051/parasite/2011183247

**Published:** 2011-08-15

**Authors:** J.-C. Beaucournu, K. Wells

**Affiliations:** 1 Laboratoire de Parasitologie médicale, Faculté de Médecine et Institut de Parasitologie de l’Ouest 2, avenue du Professeur Léon Bernard 35043 Rennes Cedex France; 2 Biodiversty and Climate Research Centre (BiK-F) Senckenberganlage 25 D-60325 Frankfurt am Main, Allemagne; 3 Sabah Parks Kota Kinabalu Sabah Malaysia

**Keywords:** *Gryphopsylla jacobsoni segregata*, *Gryphopsylla segregata*, clé du genre *Gryphopsylla*, *Gryphopsylla jacobsoni segregata*, *Gryphopsylla segregata*, key to the genus *Gryphopsylla*

## Abstract

La collecte d’une femelle de *Gryphopsylla jacobsoni segregata* Beaucournu & Sountsov, 1999 du nord de Bornéo nous permet de modifier le statut de ce taxon en celui de bonne espèce, proche mais distincte de *G. jacobsoni* (Jordan & Rothschild, 1922). La femelle de *G. segregata* est décrite et une clé remise à jour de ce genre est donnée.

## Abstract (en)

The recent collection of a female of *Gryphopsylla jacobsoni segregata* Beaucournu & Sountsov, 1999 from northern Borneo allowed us to better clarify the taxonomic status of this flea and confirm that *G. jacobsoni* and *G. segregata* are distinct species. We describe the female of *G. segregata* and revised the identification key of the genus.

## Introduction

Le genre *Gryphopsylla* Traub, 1957 est essentiellement indo-mélanésien avec un taxon dans le sud-Vietnam (Beaucournu & Sountsov, 1999). L’histoire taxonomique de ce genre est assez complexe et nous la résumerons ici. En 1957, Traub crée le sous-genre *Gryphopsylla* pour le taxon “*hopkinsi*” qu’il décrit. En fait, ce sous-genre est instauré pour une espèce montrant un “bec” en avant de la capsule céphalique. Mardon (1978) élève au rang générique *Gryphopsylla*, et en 1981, dans sa monographie sur les Pygiopsyllidae, ce même auteur inclut deux autres espèces dans ce genre, espèces sans “bec” céphalique : *G. mjoebergi* (Jordan, 1926) et *G. jacobsoni* (Jordan & Rothschild, 1922). Par ce même auteur, les genres ou sous-genres *Migrastivalius* Traub, 1980 et *Destivalius* Traub, 1980 sont placés en synonymie de *Gryphopsylla*.

Depuis la parution de cette monographie, trois taxa inclus d’emblée dans ce genre ont été décrits : *G. hetera* Lewis & Jones, 1985, *G. jacobsoni segregata* Beaucournu & Sountsov, 1999 et *G. maxomydis* Durden & Beaucournu, 2006, cette dernière seule montrant un “bec”.

La description de la sous-espèce “*segregata*” montra, à partir des mâles, d’une part, l’existence au Vietnam du genre *Gryphopsylla* et, d’autre part, officialisa l’opinion, discrètement formulée, de Mardon (1981), suggérant que le matériel à sa disposition renfermait plus d’une espèce (*cf*. carte 1). Le taxon-type de Sumatra (deux femelles, dont Jordan & Rothschild, 1922 ont dessiné le sternite VII), retrouvé à Java, était différent de celui de Bornéo. Il en est de même pour ceux de Malaisie (Cameron Higlands, Gunung Balu Brinchang), deux mâles étudiés par Traub (1950). Les spécimens du Vietnam (quatre mâles) sont indifférenciables de ceux de Bornéo et, par conséquent, se séparent de ceux de Sumatra, de Java et de Malaisie. Le sternite VII de la femelle, figuré par Mardon (*op. cit.*), mais il n’en précise pas l’origine (Sumatra ou Java ?), est plus ou moins identique à celui donné par Jordan & Rothschild (*op. cit.*). En effet, les contraintes demandées aux dessins ont évolué entre temps ! Quoi qu’il en soit, le contour de ce sternite est bien différent de celui de notre femelle de Bornéo et justifie donc l’élévation au rang de “bonne espèce” des spécimens de cette île et, par suite, de ceux du Vietnam.

**Carte 1.**
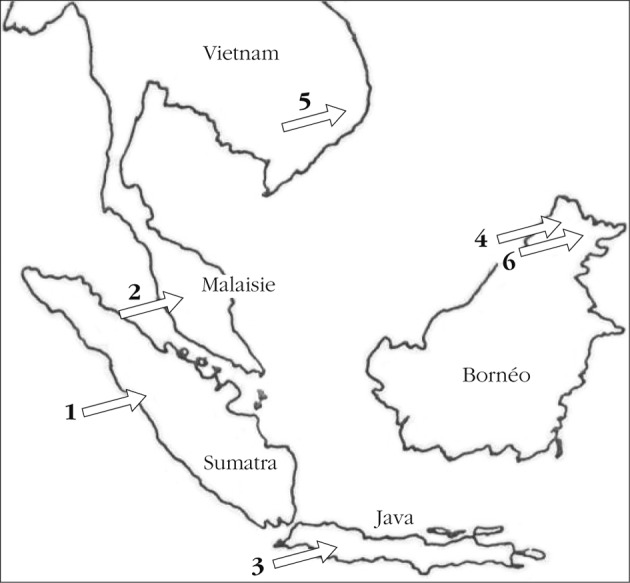
Stations connues de *Gryphopsylla jacobsoni* (1 – 2 – 3) et de *G. segregata* (4 – 5 – 6).

En résumé, le matériel ayant fait l’objet d’une publication et concernant *G. jacobsoni* s.s. est le suivant : Sumatra, deux femelles-types; Malaisie, deux mâles; Java, un mâle et deux femelles.

## Résultats

### *Gryphopsylla Segregata* Beaucournu & Sountsov, 1999

#### • Synonymie

*Gryphopsylla jacobsoni* (Jordan & Rothschild, 1922) *in* Mardon (1981), le mâle de Bornéo; *Gryphopsylla jacobsoni segregata* Beaucournu & Sountsov, 1999, mâles du sud-Vietnam.

#### • Matériel concerné

Bornéo : un mâle *in* Mardon, 1981, Mont Kinabalu, Dalas (près de Nabulu, à 10 ou 20 km de Mesilou), Sabah, sur *Rattus rattus*, 7. VIII. 1951, PAW & DJ *rec*.; quatre mâles, Mesilou, Kinabalu Park, Sabah, sur Tupaia montana Thomas, 1892, 10. XII. 2009, K. Wells *rec*.; une femelle, même endroit et même hôte, 8.I. 2010, K.W. *rec*. (Mesilou, 06°00’ N, 116°35’46 E); ces puces ont été collectées dans le cadre d’une étude des petits mammifères et de leurs puces dans deux régions montagneuses de Sabah (Wells *et al.*, 2011). Vietnam : quatre mâles (dont l’holotype de *G. jacobsoni segregata* B. & S., 1999), Pic Langbian, Dalat, sur *Chiropodomys gliroides* (Blyth, 1856), 9 et 12. VI. 1997, V.V. Sountsov *rec*.

#### • Description

**Figures 1-2.**
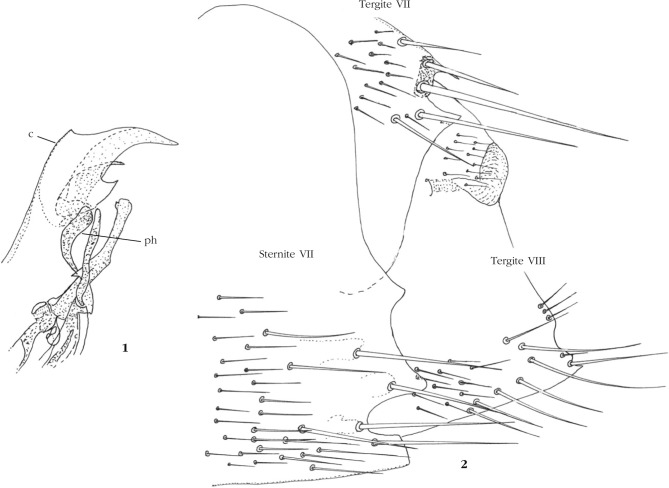
Phallosome de l’holotype de *Gryphopsylla jacobsoni segregata* Beaucournu & Sountsov, 1999, c : capuchon; ph : *phylax*. 2. Segments terminaux du “neallotype” de *G. segregata*, tergites VII et VIII, sternite VII.

Nous ne reviendrons pas sur le mâle brièvement décrit par Beaucournu & Sountsov (*op. cit.*); nous rappelons que le critère majeur de différentiation est constitué par le phallosome (figure 1) : le *phylax* (*sensu* Traub, 1980) et, à un moindre degré, le capuchon, sont nettement différents de ceux de *G. jacobsoni*.

Femelle : nous indiquerons les principaux caractères divergents entre *G. segregata* et *G. jacobsoni*. Palpe labial plus court que la coxa I. Tergite VII (figure 2) montrant un lobe long entre les soies antesensiliales droite et gauche; de même, en dessous de ces soies, le lobe est plus prononcé que chez “*jacobsoni*”. La plus haute soie latérale, en dessous des soies antesensiliales, est plus longue que la soie antesensiliale la plus haute (ce caractère valable pour “*jacobsoni*” est encore plus marqué chez “*segregata*”). Sternite VII avec un lobe concave median, celui-ci doté d’une excroissance médiane (évoquant un peu “*hopkinsi*”), inexistante chez “*jacobsoni*” chez qui, d’ailleurs, le lobe est moins ouvert. Le tergite VIII montre un apex apico-ventral très acuminé. Spermathèque : comme chez “*jacobsoni*”, *hilla* relativement longue, ne pénétrant pas dans la *bulga; ductus communis* non décrit chez “*jacobsoni*”.

#### • Discussion

Étant donnée la grande homogénéité de morphologie chez les Stivaliinae, il est normal que les critères énumérés ci-dessus semblent discrets. Toutefois, ceux-ci sont plus que suffisants pour attester de la validité de *G. segregata*. La répartition de cette espèce est indo-malaisienne et déborde sur la région orientale (sous-région indo-chinoise). Cette dispersion un peu insolite a été examinée par Beaucournu *et al*. (2000) qui estiment que l’on peut y voir un souvenir du rattachement de Bornéo au continent indo-chinois lors de la dernière glaciation (Birot, 1970).

### Clé Des Espèces De *Gryphopsylla*

1. Capsule céphalique montrant un évident “bec” préoral............................................................................... 2
  - Capsule céphalique sans “bec” préoral .................. 3
2. Présence d’un cténidie sur le segment abdominal II ....................................................................... *hopkinsi*
  - Présence de trois cténidies sur les segments II-IV ... ..................................................................... *maxomydis*
3. Présence de deux cténidies, placées sur les segments II-III .................................................... *mjoebergi*
  - Présence d’une seule cténidie, placée sur le segment II ................................................................................... 4
4. Soies spiniformes présentes jusqu’à l’apex du sternite IX ; apex du phallosome acuminé montrant une démarcation entre le “capuchon” et la longue et fine expansion de celui-ci; femelle avec un sinus sur la marge du sternite VII ................................................... 5
  - Soies spiniformes absentes de l’apex du sternite IX ; apex du phallosome acuminé sans démarcation visible entre le “capuchon” et son expansion; femelle inconnue ....................................................................... *hetera*
5. Démarcation nette entre le “capuchon” et son expansion; *phylax* en boomerang (ses deux extrémités sont identiques); femelle présentant une échancrure simple sur la marge du sternite VII ......................... *jacobsoni*
  - Démarcation peu nette entre le “capuchon” et son expansion; *phylax* plus étroit à son extrémité antérieure (ou inférieure); femelle présentant une échancrure avec une expansion médiane .............. *segregata*
